# Housing tenure and disability in the UK: trends and projections 2004–2030

**DOI:** 10.3389/fpubh.2023.1248909

**Published:** 2024-01-04

**Authors:** Michael Murphy, Emily M. D. Grundy

**Affiliations:** ^1^Department of Social Policy, London School of Economics and Political Science, London, United Kingdom; ^2^Population Research Unit, Faculty of Social Sciences, University of Helsinki, Helsinki, Finland; ^3^Institute for Economic and Social Research, University of Essex, Essex, United Kingdom

**Keywords:** housing tenure, disability, housing policy, housing and public health, inequality, United Kingdom

## Abstract

**Introduction:**

Housing is a major influence on health. Housing tenure is associated with housing conditions, affordability, and security and is an important dimension of housing. In the UK there have been profound changes in both housing conditions and the distribution of households by tenure over the past century, that is during the lifetimes of the current population.

**Methods:**

We firstly reviewed and summarise changes in housing conditions, housing policy and tenure distribution as they provide a context to possible explanations for health variations by housing tenure, including health related selection into different tenure types. We then use 2015-2021 data from a large nationally representative UK survey to analyse associations between housing tenure and self-reported disability among those aged 40-69 controlling for other socio-demographic factors also associated with health. We additionally examine changes in the association between housing tenure and self-reported disability in the population aged 25 and over in the first two decades of the 21st century and project trends forward to 2030.

**Results:**

Results show that associations between housing tenure and disability by tenure were stronger than for any other indicator of socio-economic position considered with owner-occupiers having the best, and social renters the worst, health. Differences were particularly marked in reported mental health conditions and in economic activity, with 28% of social renters being economically inactive due to health problems, compared with 4% of owner-occupiers. Rates of disability have increased over time, and become increasingly polarised by tenure. By 2020 the age standardised disability rate among tenants of social housing was over twice as high as that for owner occupiers, with projections indicating further increases in both levels, and differentials in, disability by 2030.

**Discussion:**

These results have substantial implications for housing providers, local authorities and for public health.

## Introduction

Housing has long been recognized as an important influence on individual and public health (1, 2) and numerous studies have demonstrated links between health and housing conditions, such as overcrowding, access to amenities, and exposure to a range of pathogens and risks including inadequate heating, poor ventilation, mold, trip hazards and noise and air pollution (3–7). Suitable housing is especially important for people with disabilities and older people who on average spend longer at home and may have mobility or other limitations, including poorer thermoregulation, making them more vulnerable to the effects of cold and risks posed by poor accessibility and trip hazards (8, 9).

The ways that housing may influence health are particularly broad, including social, economic, and cultural factors as well as housing conditions (2) but these other aspects of housing relevant to health, have attracted less attention than housing conditions. An important dimension of housing is tenure (whether the home is owner-occupied or rented, and if rented whether from a public or private landlord). Pathways to different housing tenures are influenced by health-related factors such as income, wealth, and family background and indeed many UK studies, including the influential Black report on health inequalities, have used housing tenure as an indicator of socio-economic position (SEP), especially for the older population for whom other indicators such as occupation or educational level are either unavailable or variation between sub-populations is limited, for example, very high proportions may have no formal educational qualifications (10–12). This selection effect complicates identifying specific effects of housing tenure on health and comparisons between countries and time periods.

However, studies from a wide range of advanced economies, for example, Japan (13), China (14), Australia (15), US (16, 17), Finland (18), and Germany (19) as well as the UK, have found that owner-occupiers have better health and lower mortality than tenants even after control for other indicators of SEP.

There have also been a number of reviews on the relationship of housing and health, some including papers on housing tenure and health, from a number of different disciplinary perspectives including: Public health: (2, 16, 20–24); Environmental health: (6, 9, 15, 25–29). Other disciplinary-orientated studies include (30–34).

A number of these studies, especially those in the environmental health category, have concentrated mainly on physical housing conditions, especially on the effect of cold homes and damp on the probability of respiratory diseases and factors such as overcrowding and lack of access to amenities.

Some comparative European studies suggest that differentials in health by housing tenure are particularly marked in the UK. Dalstra et al. (35), for example, analyzed differentials in less than good self-rated health among people aged 60–79 in ten European populations and found that, after adjustment for income and educational level, large tenure differences were evident in England and the Netherlands, although not in the other eight populations considered. More recently Acolin (36) examined differences between owner occupiers and tenants in reported poor health and depressive symptoms using EU-SILC data for 25 European countries. In analyses which controlled for a range of confounders (income, employment status, marital status, degree of urbanization), he found that that differences by housing tenure were widespread, and differences between owners and renters in depressive symptoms were largest in the UK. There is also evidence that tenure differentials in mortality in the UK increased in the late 20th and early 21st centuries (37–39). This may reflect major changes in policy, notably the mandated sale of public housing to tenants at discounted prices introduced in 1980 which led to large changes in the distribution of households by tenure (40) and possibly to increased societal inequalities.

Several explanations, not mutually exclusive, have been proposed to account for these associations between housing tenure and health net of other indicators of SEP. Firstly, it has been suggested that there may be health related selection factors into tenure types not fully accounted for by control for other indicators of SEP, for example family background and childhood circumstances (18). However a recent study of three British birth cohorts found higher premature mortality among tenants compared with owner occupiers even after control for a very wide range of childhood, as well as adult, factors (41). Differences by tenure in health-related behaviors, such as smoking, may also be relevant (42), although some UK studies which have controlled for this have still found variations in health by tenure (43).

In addition to selection issues, several studies have suggested that tenure differences in internal housing conditions and neighborhood characteristics may mediate associations between housing tenure and health (7, 19, 44, 45). This may be a particular issue in the UK as the proportion of housing dating from pre 1946 is higher than in any EU member state (46) and much of the housing stock needs renovation (47). Tenure may also be associated with affordability, and so with financial stress which has consequences for mental health (48, 49). Additionally psychosocial influences related to perceived status, residential stability and ontological security may be important (36, 45, 50) and may vary by time and location.

Regardless of the factors underlying tenure differences in health, knowledge of the extent of variation is important for housing providers and policy makers concerned with targeting resources and delivering services (43). Much of the discussion about housing has focused on the increasing difficulties faced by those by those entering the system, mainly young people, for whom the growing unaffordability of home ownership and reduction in the stock of social rented accommodation has forced reliance on the privately rented sector (51). This sector is now characterized by short insecure contracts, uncontrolled rents, and poor housing quality (52) which studies suggest have adverse implications for mental health, although longer term implications for health are as yet unclear (53). With some exceptions, the situation of those in mid-life and older age groups has received less attention (47).

Changes in housing policy over time may have led to increasing health differences between those in different tenures, which is the main focus of this paper. Our main health indicator is self-reported disability, which has increased in prevalence in recent decades. This trend has become of increased concern, not only from a public health perspective, but also because of the economic effects of a shortfall of 500,000 older workers who did not return to the labor force after the Covid pandemic (54) resulting in shortages of workers in some key sectors and contributing to lower economic growth and higher inflation. This has been linked to health-related issues, and housing might be expected to be implicated here as well. For these reasons, we give particular attention to the relationship between the housing and health of those aged 40–69.

In the UK, both housing conditions and the distribution of households by tenure have undergone profound changes over the past century. These are reviewed and summarized below as they provide a context to possible explanations for health variations by housing tenure, including health related selection into different tenure types and variations in this by age and cohort.

### Housing policy, housing tenure and health: the UK context

The UK has three main housing tenures; owner-occupied housing (either owned outright or being purchased with a mortgage); what is now termed ‘social housing’ rented from a local authority or a not-for-profit housing association or trust; and privately rented housing. In the past distinctions between those renting unfurnished or furnished privately rented accommodation were important as the former had more security of tenure, but since 1988 both groups have been equally insecure.

There have been major changes in the distribution of housing by tenure over the past century. The provision of public housing for rent, for example, expanded from 1920 until the 1980s and then contracted with social housing consequently shifting from a mainstream to an increasingly residualized tenure (55). Currently there is an acknowledged housing crisis with problems of housing supply, housing affordability and housing quality which have differentially affected population subgroups (47, 56, 57).

Mullins & Murie (58) identified two 20th century turning points in UK housing policy and provision which had substantial effects on housing supply and the distribution of housing by tenure throughout the 20th and into the 21st centuries. The first of these was during World War One and its aftermath when in response to appalling housing conditions, housing shortages, and rent strikes two major housing policy initiatives were introduced. Firstly, rent controls for privately rented accommodation, at that point the tenure for three-quarters of households^1^ (60) were introduced in 1915. This was planned as a temporary measure but rent controls of one kind or another remained in force until 1988 (61). Secondly, the 1919 Housing and Town Planning Act required local authorities to provide housing for the working classes, the ‘homes fit for heroes’ promised by Prime Minister Lloyd George for soldiers returning from the First World War. These were homes of high standards (for the period) and indeed rents were too high for the poorest and the dwellings catered mainly for the better-off and aspirational working class (55, 62). Following the 1919 Act, and subsequent, legislation, council house building grew in the inter-war period (63) and expanded even more rapidly in the post-World War Two period when there was a pressing need to replace the homes destroyed or damaged by war time bombing. New construction by councils peaked in 1954 but continued apace in the 1960s and 1970s and by 1979 local authority housing comprised 31% of homes in England (55, 64). Building of housing for owner-occupation also expanded considerably throughout the twentieth century and by 1979 this sector constituted just over half of the total stock. The size of the privately rented sector and its share of the total fell sharply from the 1920s reaching a low point of 9% around 1990, but has since grown rapidly.

These recent changes are linked to the second major turning point in 20^th^ century housing policy identified by Mullins & Murie (58), the 1980 Housing Act which gave local authority tenants to right to buy their homes at very substantial discounts. Over 100,000 properties were sold in each of the three financial years following this legislation (65), sales subsequently fell but rose again between 1988 and 1990.^2^ The ‘Right To Buy’ was abolished in Scotland (2014) and Wales (2018) but remains in force in England. By 2017 over 40% of former council homes in England were in the private sector (65) and the legislation was primarily responsible for an increase in the share of homeowners among UK householders from 55% in 1979 to over 70% in the early 2000s (40). An important additional factor was the collapse in building of council and other social housing following the Act, as local authorities were not allowed to use income from sales for new construction (64). Within the social rented sector, the sharply restricted public funding was concentrated on housing associations and some local authorities transferred their stock to housing associations, in part because tenants did not have the right to purchase such properties. The 1980 Right To Buy legislation thus had major implications for the distribution of households by tenure, and their characteristics. However, even prior to this a range of policies had been introduced to promote owner occupation as the preferred tenure, including tax incentives, and shift allocation policies for council housing towards a welfare based, rather than a public housing model. The 1969 Cullingworth report, for example, proposed needs-based allocation models for council housing, the 1973 National Rent Rebate scheme increased access to council housing for those on very low incomes and the 1977 Housing Persons Act, which mandated local authorities to provide accommodation for certain groups of homeless people, cemented needs-based allocation policies (55).

These policy changes are highly relevant to any consideration of tenure differentials in health as they have influenced both health related selection into tenure types and the characteristics of the housing stock by tenure. Those most likely to take advantage of the large subsidies available under the Right to Buy legislation, for example, were families and older couples living in “family friendly” dwellings who had the economic security to be able to undertake the long-term commitment of a mortgage and qualified for the largest discounts due to the length of their tenure. Therefore it is likely that those who bought and so transferred to the owner-occupied sector had better health, as well as greater financial resources, than those who remained as council tenants, as shown in one study which compared the psychological health of this group with non-buyers (50). Those who remained in the social housing sector were disproportionately drawn from those with disadvantages, including lower incomes and wealth, and possibly worse health, so increasing the differential between these sectors.

#### Housing tenure and housing conditions

Tenure differences in internal housing condition and neighborhood characteristics have been proposed as a mediator of associations between housing tenure and health (19, 45) and several UK national or local studies have reported much poorer conditions in rented than in owner-occupied accommodation (7, 44). However, relative conditions, as with the distribution of housing tenure, have changed over time. As already noted, council housing was initially of a high standard for the time and included a large proportion of houses with gardens. The post-World War Two development of New Towns also prioritized building of houses. However, from the mid-1950s local authorities were also incentivized to build high rise and system-built housing sometimes prone to damp, condensation, noise, and other problems (67), although all housing had to meet minimum ‘Parker-Morris’ standards of space and amenities as set out in legislation in 1967 (rescinded in 1980). The Right to Buy legislation led particularly to sales of better-quality council housing (40) resulting in an overall deterioration in standards, but the Decent Homes Standard, introduced in 2000 with subsequent revisions, set out a timetable for improvements (about, for example, hazards and energy efficiency) in the remaining social rented sector. The proportion of homes meeting these standards is now highest in the social rented sector and lowest in the privately rented sector (64, 68). For example, in 2015–2017 among households including a person aged 60 and over, 30% in the privately rented sector were classed as non-decent overall, compared with 21% of dwellings owned outright by the occupiers and 15% in the socially rented sector (68). This standard relates to quality of housing and conditions within a dwelling and does not account for aspects of the local environment which may also be related to health. The proportion of social renters living in flats, including high rise flats, for example, is much higher than among owner-occupiers (69) and in 2004 a quarter of social housing in England was located in the 10% most deprived areas (64).

Overall, changes in policy, provision, and allocation policies in the post World War Two period imply a greater concentration of people with poor health, or characteristics related to poor health, in the now smaller socially rented sector than was the case in the middle decades of the 20th century. Whereas social housing for much of the 20th century housed those with a range of incomes, the reduction of supply and changes in policy recommendations and legislation meant that it has become increasingly residualized (55, 64). These period changes in supply, desirability, and allocation policies suggest that there may be age and cohort variations in health differentials by housing tenure.

The sharp reduction in privately rented accommodation in the post-War period due to the transfer of properties into owner occupation (including sales to tenants with accrued rights) and loss of existing buildings due to slum clearance and redevelopment meant that, in practice, this tenure ceased to be a mainstream option for families and was increasingly used as a short-term stopgap for groups such as students, other sharers, or young couples waiting to access owner occupied or socially rented housing, although there remains a small group of older tenants who took up tenancies before 1989 and have more rights with regard to rent control and security of tenure than those entering the sector at a later date (61). The simultaneous increase in social rented housing was particularly oriented towards young families, with the result that in the 1960s a much higher fraction of children were brought up in social rented housing than in either earlier or later periods (70). This group is central to our discussion of housing for contemporary older people. The reduction in social housing in the latter part of the 20th century, due both to sales under Right to Buy and the effective embargo on building of new council homes meant that these children faced a very different housing future when they came to establish their own households.

#### UK studies on housing tenure and health

Despite a renewed interest in the implications of housing and housing tenure for health (26, 71), there seem to have been relatively few recent UK national studies which have examined differentials in health by housing tenure with a focus on mid-life and older age groups. We undertook systematic searches of the literature, but in many studies housing tenure is considered as a co-variate, rather than a primary exposure of interest, which hampers identification of relevant research. Several studies based on analyses of the ONS Longitudinal Study of England & Wales (which links vital registration information to a census sample) and equivalent studies in Scotland and Northern Ireland have investigated differentials in self-reported health, long-term illness, and mortality by a range of socio-demographic indicators, including housing tenure. All these studies have found that owner occupiers have the best, and social tenants the worst, health and mortality even after adjustment for other relevant factors including occupational social class, marital status, car ownership and educational level (10, 37, 39, 72, 73). Analyses of other national longitudinal surveys have reported that social tenants have higher rates of disability, and progression of disability in early old age than owner occupiers or private tenants (74). However, Chandola (75), in analyses of data from the British Household Panel Study, found that although owner occupiers had the lowest mortality, there was no statistically significant difference between social and private renters; similarly other studies have reported that owner occupiers have the best health or quality of life but either find no difference between social and privately renting tenants, or have not been able to investigate this (76, 77). Feinstein et al. (70) investigated differences in a composite indicator of deprivation, which included some measures of health, at various ages for members of the 1946, 1958, 1970, and 2000 British birth cohort studies. For 1946 cohort members, living in social housing in childhood or at age 36 was not associated with worse later outcomes (the latest observation being at age 62) in comparison with those in the private rented sector, but in the 1958 and 1970 cohorts being in social housing in childhood or at age 30/33 was associated with later disadvantage. This suggests that, as might be expected, changes over time in the selection processes into various housing tenures are likely to be reflected in changing associations between housing tenure and health.

In this paper we use a large scale nationally representative data set to investigate associations between housing tenure and reported disability in the 21^st^ century in the UK. We firstly focus on the population aged 40–69 and analyze differentials in health by tenure taking account of other socio-demographic indicators. We secondly consider trends in associations between housing tenure and reported disability in the whole population aged 25 and over for 2004–2021 and project results forward to 2030.

## Materials and methods

We use data from the UK Annual Population Survey (APS) 2004–2021 to examine differentials in self-reported disability and long-term health problems by housing tenure, controlling for sex, age and other socio-demographic characteristics. The APS is a continuous household survey which uses data from the Labour Force Survey (LFS), together with local area sample boosts, and has the largest coverage of any household survey in the UK (78). The APS is a primary source for both official disability and household statistics and is of sufficient size to provide robust estimates of time trends in key economic variables and estimates of disability for population sub-groups. The survey aims to interview all adults aged 16 and over in sampled households, with proxy interviews collected for those unavailable at interview.

The APS has a complex design. The sample frame is of addresses and the survey includes the mainstream Labour Force Survey (LFS) in which residents at selected addresses are re-interviewed for five successive quarters. If a sample household has moved, an attempt is made to re-interview them, but otherwise the new household living at the selected address is invited to participate. The other main component of the APS is a boost sample to enable production of robust annual sub-national estimates. However, these boost addresses are reinterviewed four times annually. Not all questions are repeated in the re-interviews; importantly for this study, those aged 65 and over are generally asked about health only at the first contact (79). As we wanted comparable information for younger and older respondents, we confine analyses to responses from the first wave of each survey round.

Responses to health questions were not included in the APS files, so for Table 1, we use the Labour Force Survey component.

**Table 1:** Sample characteristics, UK Annual Population Survey, people aged 40–69, 2015–2021.

**Characteristic**	**Owned, *N* = 257,272** ^**1**^	**Private rented, *N* = 33,863** ^**1**^	**Social rented, *N* = 45,941** ^**1**^
Disability			
Yes	56,667 (22%)	10,049 (30%)	24,706 (54%)
No	199,406 (78%)	23,608 (70%)	20,944 (46%)
Unknown	1,199	206	291
Age	55 (48, 62)	50 (44, 58)	54 (47, 61)
Sex			
Male*	123,017 (48%)	17,152 (51%)	20,598 (45%)
Female	134,255 (52%)	16,711 (49%)	25,343 (55%)
HiQual			
Tertiary*	105,259 (42%)	11,054 (34%)	5,676 (13%)
Upper secondary	50,587 (20%)	5,897 (18%)	6,817 (15%)
Lower secondary	51,952 (21%)	6,870 (21%)	10,134 (23%)
Other/None	42,954 (17%)	9,165 (28%)	21,737 (49%)
Unknown	6,520	877	1,577
NS-SEC			
High*	93,141 (42%)	8,575 (29%)	4,077 (10.0%)
Medium	63,575 (29%)	8,502 (29%)	7,917 (19%)
Low	36,265 (17%)	7,706 (26%)	13,426 (33%)
Never worked, unemployed, and nec	26,724 (12%)	4,765 (16%)	15,488 (38%)
Unknown	37,567	4,315	5,033
EconAct			
In employment*	178,973 (70%)	24,057 (71%)	20,077 (44%)
ILO unemployed	3,440 (1.3%)	1,224 (3.6%)	2,082 (4.5%)
Sick/Injured/Disabled	9,528 (3.7%)	3,263 (9.6%)	12,897 (28%)
Retired	50,000 (19%)	2,409 (7.1%)	5,987 (13%)
Other (unemp, homemaker etc.)	15,331 (6.0%)	2,910 (8.6%)	4,898 (11%)
Partner			
Partnered*	208,296 (81%)	19,917 (59%)	21,699 (47%)
Not partnered	48,976 (19%)	13,946 (41%)	24,242 (53%)
Marital			
Married*	185,200 (72%)	15,716 (46%)	16,971 (37%)
Divorced/Separated	31,091 (12%)	9,327 (28%)	12,777 (28%)
Single, never married	33,006 (13%)	7,833 (23%)	13,530 (29%)
Widowed	7,975 (3.1%)	987 (2.9%)	2,663 (5.8%)

^1^*n* (%); Median (IQR); * Reference category in regressions. Source: Annual Population Survey.

### Measures

#### Outcome variable

The APS is one of the two main sources for UK official disability estimates. The main outcome measure is derived from a question on disability/health problems in the APS using responses from a large random sample of adult informants The question used was based on extensive testing taking into account the recommendations of the United Nations Washington Group (80). Prior to 2013 this question asked whether respondents had any ‘disabilities or long-term health problems.’ Disability became a protected group category for the first time in the 2010 Equality Act. Landlords, both private and public, can be legally required to make “reasonable” adaptions to property to meet the needs of disabled tenants. This necessitated a minor change to the question wording to asking whether respondents had any ‘physical or mental health conditions or illnesses’. Under the terms of this Act, people with disabilities would also be able to ask local authorities for assistance and support in applying for social housing (for example help in filling in forms) but not have a right to social housing *per se*.

The original question was retained until 2020 allowing effects of the change in wording to be assessed (80). This showed that the change in question wording resulted in a slight drop (0.9 per cent) in the proportion of economically active respondents aged 16–64 reporting a disability/long-term health problem (81). Overall results, including regression results shown later, from the new and old disability questions were extremely close. Therefore, in this study we used the responses to the original question to examine longer term trends over period 2004–2020, and the second set for detailed analysis for the more recent period, 2015–2021. We also present some results on the main reported type of health problem which has been collected in some rounds of the survey.

#### Housing tenure and other co-variates

Our main exposure variable is housing tenure. We distinguish between the three main housing tenures in the UK: those living in owner-occupied housing; social renters; and private renters. The very small remaining proportions living rent-free or renting from employers, relatives etc. were included with private renters.

Other variables used in the analysis include: level of highest educational qualification (HiQual), distinguishing between those with tertiary level qualifications, higher secondary (‘A-level’), lower secondary (GCSE/GCE ‘O level’), and other/no qualifications: occupationally-defined socio-economic class assigned using the National Statistics Socio-Economic Classification (NS-SEC) with a three-fold classification into high (professional and managerial); medium (intermediate; small employers and own account workers, and lower supervisory and technical); and low (routine and semi-routine); and an unclassified group (never worked, unemployed, and not elsewhere classified): economic activity (EconAct) with two economically active groups; employed and unemployed (ILO definition); and three inactive groups, sick/injured/disabled, retired, and other (e.g., homemaker etc.): marital status (married; divorced or separated; never married; widowed); and partnership, whether the informant is cohabiting or not irrespective of formal marital status. Numerous studies have shown that these variables are all associated with health differentials (75). Information on economic activity, marital status, sex, age, housing tenure and disability are available since 2004 for all respondents (NS-SEC is available only for those with a reported occupation and also changed in 2010), but the APS has only collected full information on highest educational level since 2015 and only for sample members aged 16–69. Some indicators such as unemployment and disability are included in multiple questions and model specifications have been adjusted to allow for this redundancy.

### Methods

#### Multivariable analysis of associations between disability and tenure among persons aged 40–69

In the first stage of analysis, we used multivariable logistic regression to assess the association between housing tenure and disability controlling for the other previously mentioned socio-economic indicators known to be associated with disability. In more detailed analyses we included interaction terms between housing tenure and these other variables to examine whether associations varied according to housing tenure. These models are confined to the years 2015–2021 and to ages 40–69 due to the data availability constraints for those aged 70 and over discussed earlier and our main focus on mid and later-life. We chose 40 as the lower age boundary as by that age the great majority (95%) live in a household headed by themselves or a spouse/partner. We also fitted a model to the data for the whole period, 2004–2020, including all variables except highest educational qualification, which is not available prior to 2015, and using the disability indicator available throughout.

#### Trends and projections of disability by housing tenure among all adults aged 25 and over

In the second stage of analysis we estimated the main trends in disability by housing tenure for all adults aged 25 and over. To do this we fitted a series of generalized additive logistic models (gam) for the period 2004–2020 and projected the results forward to 2030 to provide trend analysis. The chosen model is flexible as there is no pre-specified form for the relationship. We fitted separate models to the three tenure groups. The total sample size was 1.8 million.

#### Weighting

The APS includes weights designed to gross up sample values to match official statistics. These account for the different designs of the APS components, multiple interviews and attrition between waves, but relate to the full all-wave APS sample and are not appropriate for this study. In addition, the statistical approach used here, generalized additive models, does not have the option of including full design weights, although influence rather than sample weights can be included. For these reasons, the results presented in this paper are unweighted.

There is a substantial literature about the pros and cons of weighting: clearly unweighted or inappropriately weighted data should not be used to gross up results and provide population estimates, but methodologists differ on whether weights should be used in regression models (82). We undertook some sensitivity analysis comparing weighted and unweighted results for the full multi-wave APS sample. The results for confidence intervals are based on standard practice where weights are treated as influence rather than sample weights. Detailed sample design variables such as on primary sampling units (psus) and stratification are not available even in the restricted publicly available datasets, but analyses show that design effects for the types of variables shown here are in the range 1.1–1.2 (83). Although this implies that the nominal 95% confidence intervals shown here probably slightly understate those that would have been obtained with full sample design information, we concluded that results of weighted and unweighted analyses are almost identical.

## Results

### Sample summary

Table 2 presents descriptive results for the main analysis sample of people aged 40–69 pooled over the 2015–2021 rounds of the APS. There were substantial differences between tenure groups in characteristics known to be associated with health with owner-occupiers being considerably more advantaged in terms of education, occupation, and partnership status than renters (we report precision estimates in the multivariate analyses later). These differences likely reflect life course differences in financial ability to purchase a home and socio-economic and health related influences on transfers between tenures. For example, historically, a major reason for tenure change has been movement from the owner occupied to the rented sector, especially the social rented sector, by women with dependent children experiencing relationship breakdown and lacking the resources to purchase a property.

**Table 2:** Distribution by economic activity and disability status, people aged 40–59, UK, 2015–2021.

	Owner occupier	Private renter	Social renter
In employment	86.5	77.7	53.3
*Of which:*			
*In employment, not disabled*	*73.9*	*63.8*	*39.5*
*In employment, disabled*	*12.7*	*13.9*	*13.8*
Unemployed (ILO definition)	1.6	3.8	5.5
Inactive because sick/injured/disabled	3.2	8.9	28.2
Retired	2.5	0.5	0.5
Other unemployed, homemaker etc.	6.1	9.0	12.5
Total sample (=100%)	166,422	26,923	31,315

Source: Annual Population Survey.

Differences between tenure groups in economic activity were particularly marked, with 4% of owners reporting that they were economically inactive for health reasons compared with 28% of those in the social rented sector. Consistent with this finding, the proportion of social tenants reporting a disability was, at 54%, nearly two and a half times the equivalent for owner occupiers (22%). Private renters were generally intermediate across the variables shown, apart from age, where they are younger on average. They were also much less likely to have been living at their current address for an extended period than those in other tenure groups. Although length of residence has been associated with health differences (36), in preliminary analyses, we found that neither length of residence nor ethnicity contributed to explaining observed differentials in disability by tenure so these variables were not included in subsequent analyses.

### Multivariable analysis of differentials in disability among those aged 40–69

Given these differentials between groups, the question arises as to how far differences in disability levels between tenure groups reflect compositional effects rather than factors associated with housing tenure itself. We analyzed tenure differentials in disability, taking account of differences in other health-related characteristics, using multivariable analyses including all the variables shown in Table 2, except the partnership indicator which in these age groups overlaps substantially with marital status. We specify age in quadratic form.

**Figure 1:**
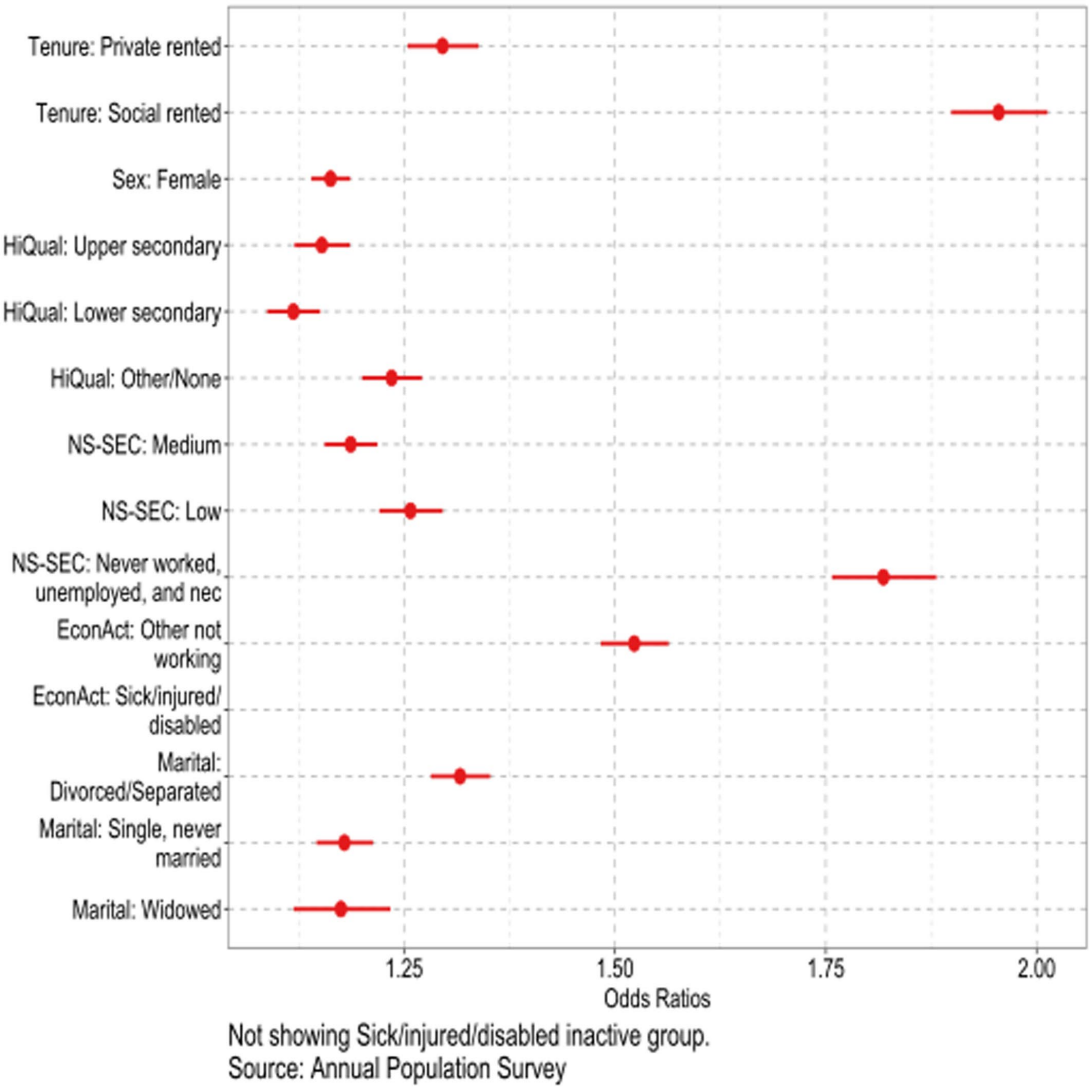
Results from logistic regression for disability; United Kingdom, people aged 40–69, years 2015–2021.

We first present results from logistic regression analysis showing the odds ratios of reporting disability (Figure 1) using the data shown in Table 2. Full parameter estimates and estimates of precision are given in Supplementary Table S1. The reference categories were chosen to have the smallest effects on the probability of disability (see Table 2). We used the full sample, including those who reported themselves as economically inactive for health reasons, but we do not show this coefficient, since almost all of this group reported that they were currently disabled. However, they were included in the regression model since they form a particularly large fraction of the social renting population and contribute to the magnitude of differences between tenure groups. Since economic activity is often a consequence rather than a cause of disability, we discuss employment in more detail later. Being unmarried (Divorced/separated OR 1.33, s.e. 0.02), having low educational qualifications (Other/none OR 1.24, s.e. 0.02) or occupational class (Never worked, unemployed or not elsewhere classified OR 1.82, s.e. 0.03; Routine and semi-routine OR 1.26, s.e. 0.02) and living in rented accommodation (Social rented OR 1.95 s.e. 0.03; Private rented OR 1.29, s.e. 0.02) were all associated with particularly high odds of reported disability. Notably, when these variables were all included in the same model, the largest reported odds ratio was for social renters as compared with the reference group of owner-occupiers. With these controls, this coefficient was 1.95, as compared with 4.47 (s.e. 0.05) in a model controlling only for age and sex (see Supplementary Table S1 for full details), reflecting the substantial socio-demographic differences between tenure groups, but also indicating that housing tenure had a considerably stronger influence on disability than widely-used socio-demographic variables such as education, occupational class or partnership.

#### Predicted values among population sub-groups

**Figure 2:**
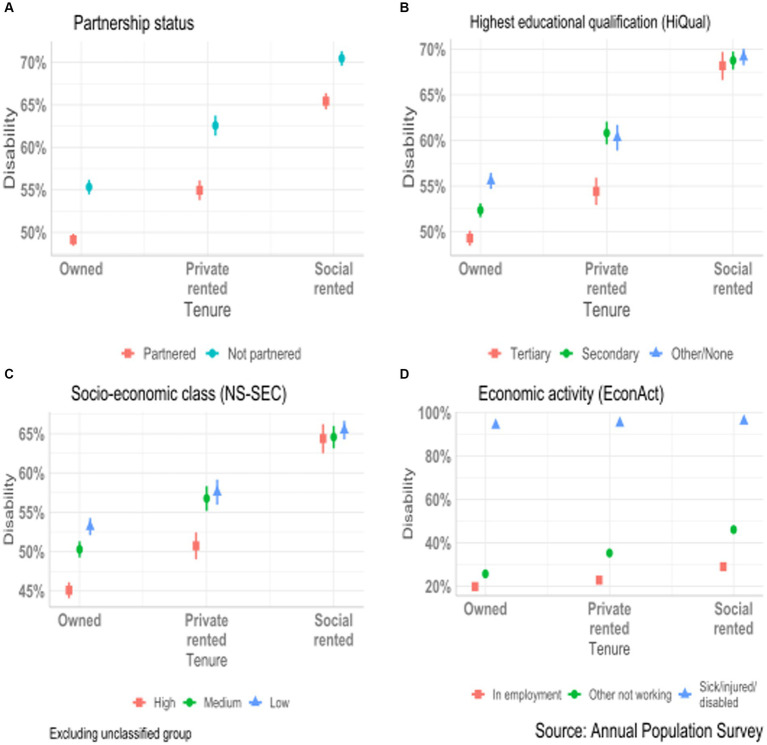
Conditional predicted probability of disability for United Kingdom population aged 40–69 in years 2015–21 by housing tenure and selected socio-demographic variables: **(A)** Partnership status. **(B)** Highest educational qualification. **(C)** Socio-economic class. **(D)** Economic activity.

Since educational level, occupational class, and partnership status are strongly linked to both health status and housing tenure, we undertook more detailed analyses to examine if the relationships shown in Figure 1 are similar across tenure groups. The three models presented were identical to that of Figure 1 apart from including an interaction between housing tenure and each of these three variables. To simplify presentation, we reduced the number of categories. We used partnership rather than marital status; combined the two central educational categories since the coefficients in the combined groups were similar; and excluded those who were unclassified in the NS-SEC analyses to concentrate on social class differences. We present these results as marginal predicted values (i.e., predicted values for selected variables shown, while averaging over the remaining variables included in the analysis) to show the implications at the population level (Figure 2, full parameter estimates and estimates of precision are given in Supplementary Table S2). The relationship between disability and housing tenure remains strong, when educational level, NS-SEC and partnership status are included. Differences between tenure groups are larger than those between the sub-groups of the other variables; for example, the predicted probability of being disabled is 23% greater for owners than for social renters averaged across the three educational groups in Figure 2, whereas the predicted probability of being disabled is 9% higher for those in the other/none education group than those in the tertiary education group when averaged across the three tenure groups. The patterns within each tenure group are generally in the expected direction especially for owners, although education and occupational differences are negligible for social renters. These results confirm that in the UK, housing tenure has a stronger relationship with disability than these more commonly used indicators of socio-economic position and that applying separate controls within each tenure group has only a limited role in modifying differences between tenure groups.

Labor market status is strongly affected by health status, while there is no such obvious direct mechanism linking variables such as educational level or housing tenure with disability Reported disability is the major reason for the difference in economic activity between tenure groups. Table 3 shows values for the pre-retirement age group, those aged 40–59. The proportions not in employment range from 13% in the owner occupied to 47% in the social rented sector. This is mainly because 25% more social renters than owners reported health-related economic inactivity. The relationship between disability and economic activity was much weaker among those in the owner-occupied sector with 70% of those who reported a long-standing health problem being in employment compared with 28% of those in the social rented sector.

**Table 3:** Standardized proportion (%) reporting main health problem, people aged 40–69 by housing tenure, UK, 2020.

Main cause^1^	Owner occupier	Private renter	Social renter
Joint	7.6	6.5	13.5
Circulatory/respiratory	8.8	6.7	9.5
Mental and behavioral disorders	5.2	9.4	16.3
Other	12.5	10.8	15.9
None reported	65.9	66.7	44.8
Sample size (=100%)	145,922	28,328	23,904

^1^Joint: problems or disabilities (including arthritis or rheumatism) of arms, hands, legs, feet, back and neck. Circulatory/respiratory: chest or breathing problems, asthma, bronchitis, heart, blood pressure or blood circulation problems. Mental and behavioral disorders: depression, bad nerves or anxiety, severe or specific learning difficulties, mental illness or suffer from phobias, panics or other nervous disorders, autism (including autism spectrum condition, Asperger syndrome). Other: difficulty in seeing or hearing, speech impediment, severe disfigurements, skin conditions, allergies, stomach, liver, kidney or digestive problems, diabetes, progressive illness not included elsewhere other health problems or disabilities. Source: UK Labour Force Survey, 2020, standardized using ESP2013.

Potential explanations might include the fact that those living in rented accommodation are more likely to have had manual occupations, and so perhaps be less able to continue working and therefore take early retirement and report this as the reason for inactivity. Conversely those with professional jobs may be able to continue working even if they have some health limitations and may additionally have a stronger financial incentive to do so. The propensity to report disability may also differ between tenure groups.

Responses to health questions were not included in the APS files, so for this Table, we use the Labour Force Survey component (Table 1). The detailed causes asked about have been collapsed into four groups. The age standardized proportions of people aged 40–69 reporting problems were about one third among owner occupiers and private renters, but 55% among social renters. Social renters had the highest prevalence of problems in all of the major cause groups shown, with particularly high values for reported mental and behavioral health conditions, over three times the value for owner-occupiers.

### Trends in self-reported disability by housing tenure among those aged 25 and over, 2004–2020 and projections to 2030

We now consider trends in in self-reported disability and tenure over for the period 2004–2030. We extend the age range to all adults aged 25 and over since these younger people will be entering the 40 and over age group within the next 15 years. These estimates were derived from smoothed estimates of underlying trends using a generalized additive model fitted to the age by period prevalence of disability in each tenure group over the period 2004–2020 (see Supplementary Figure S1):


$$\mathrm{logit}\left(d_{\mathrm{apt}}\right)=s\left(a,p\right)*t+e_{\mathrm{apt}}$$

where *d_apt_* is probability of disability for a person aged *a* at time period *p*, living in housing tenure type *t.*

*s*(a,p) is a smoothed non-parametric surface, with an interaction with housing tenure, so that separate models are fitted to each tenure group. *e*_apt_ is a residual term error term.

**Figure 3:**
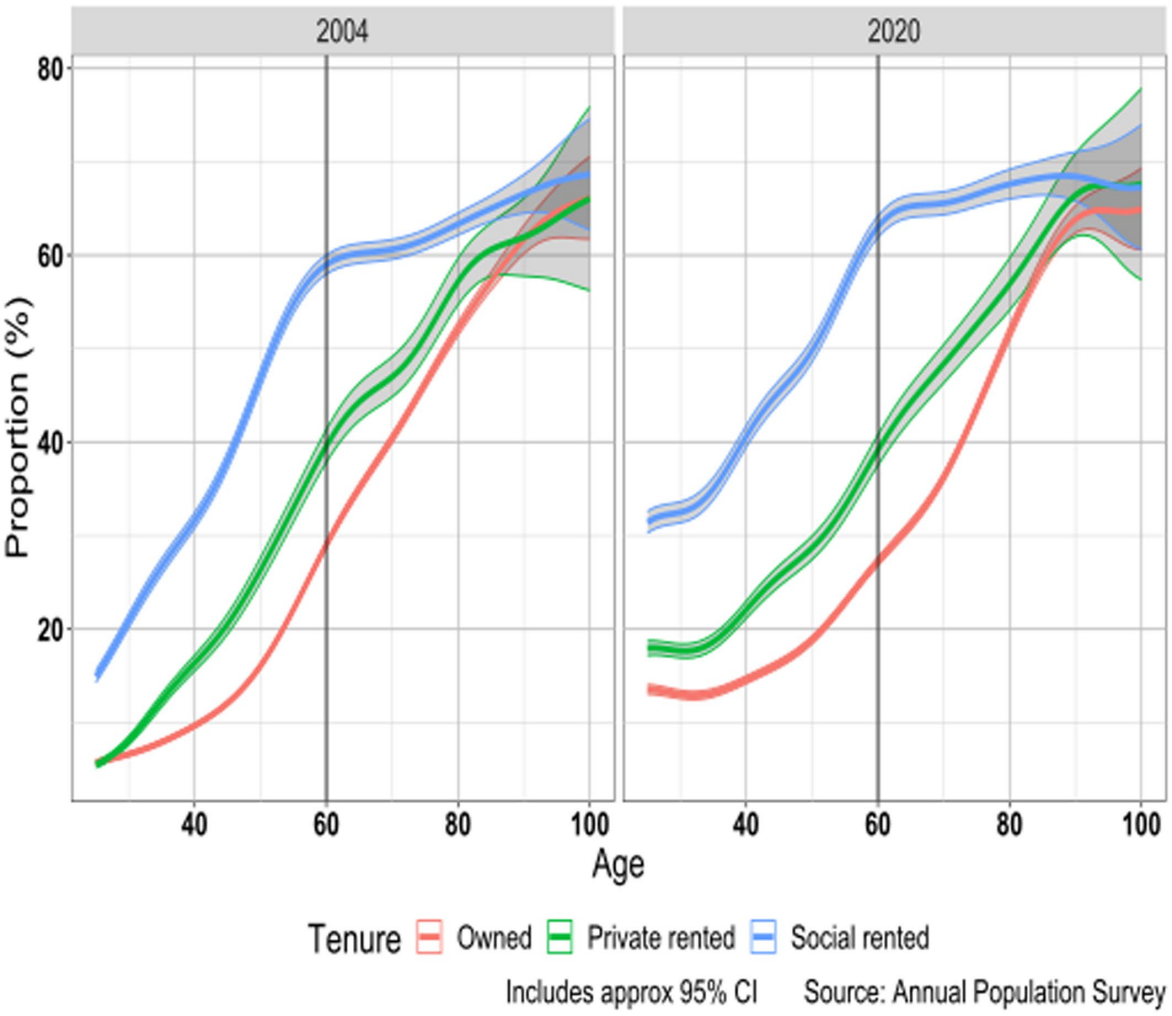
Proportion (%) disabled by age and housing tenure; United Kingdom 2004 and 2020.

We then projected these observed disability trends to 2030 to show what would happen if current trends were to continue. Figure 3 shows the proportion disabled by age and tenure for 2004 and 2020. In both years the proportions disabled were markedly higher among social renters than either private renters or owner occupiers, with the latter having the lowest rates. Reported disability was higher in 2020 than in 2004 with larger increases among younger than older adults and among renters than owners. For example, at age 40 in 2004, the estimated proportions disabled were 10, 16, and 32% in the owner occupied, private rented and social rented sectors; by 2020, these had increased to 15, 22 and 41%. By 2030 if present trends continue, these figures would be 22, 31 and 51%.

There is a non-linear trend of reported disability with age in all tenure groups with an increase in disability with age which slowed down around age 60. Differentials between tenure groups increased up to around age 60 and the largest absolute differences by tenure are found around this age when in 2020 63% of social renters reported disability compared with 27% of owner-occupiers and 39% of private renters. After age 60 differentials decreased and by the oldest ages were considerably diminished. This variation in differentials between tenure groups by age may reflect cohort differences in the extent to which social renters have characteristics associated with poor health (for example greater priority given to health problems in allocation decisions in later cohorts) and possibly tenure variations in moves into residential and nursing homes as some studies have found that these are more common among social renters than owner occupiers (39, 84).

**Figure 4:**
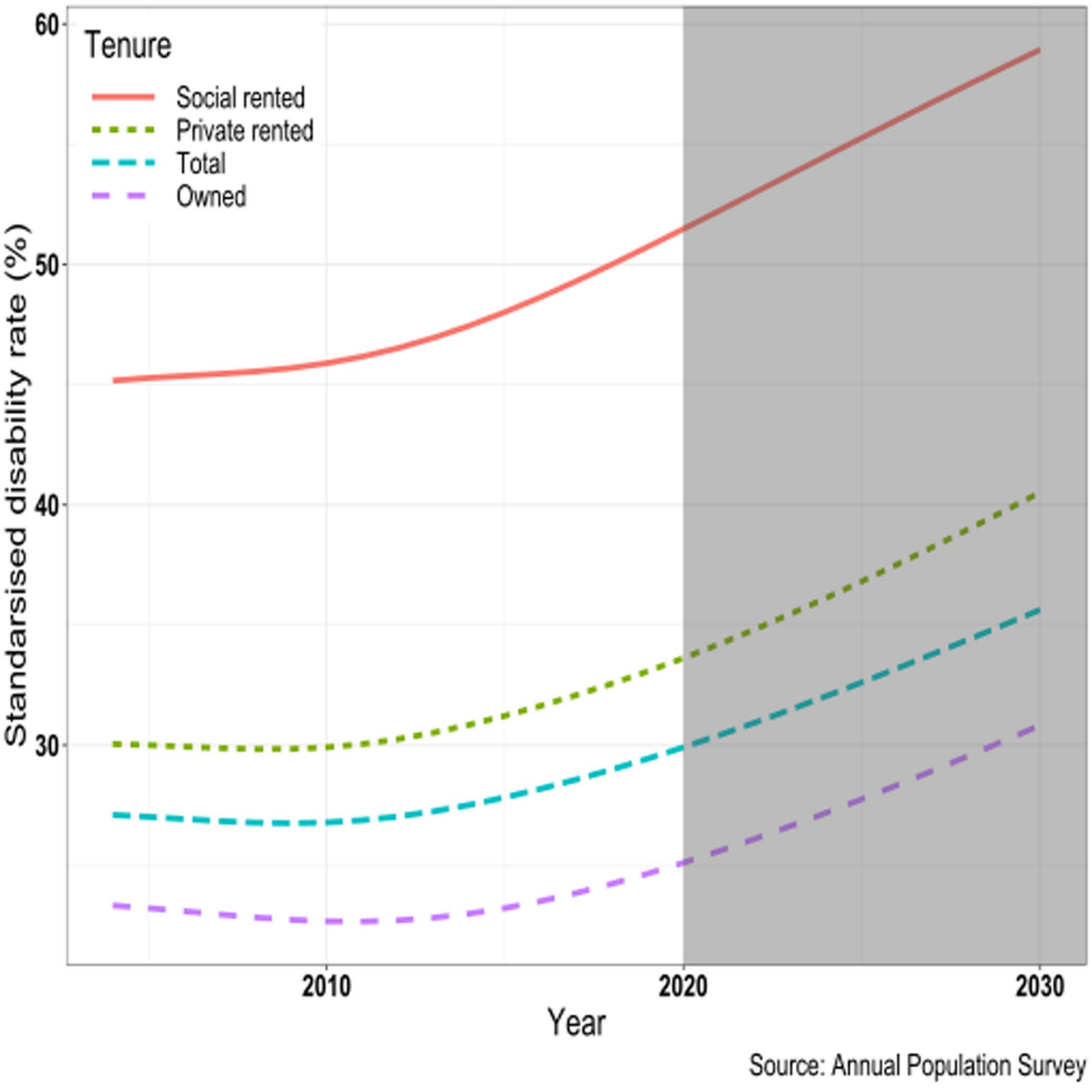
Age-standardized disability rate (%) by housing tenure: trends and projections, United Kingdom, 2004–2020, ages 25 and over.

The particularly rapid increases in reported disability among younger adults has implications for future population health as these groups age. To illustrate these time trends more clearly, in Figure 4 we present age-standardized rates of disability calculated using the European Standard Population (ESP2013) for people aged 25 and over. This shows that there was little change in overall levels of disability in the period up to 2010, but disability increased subsequently, and the increase was greater in the second half of that decade than in the first half for all tenure groups. This change in trend around 2010 occurred around the same time as there was a sharp trend change in mortality that reversed the substantial improvements observed in the first decade of the century (85). While trends for all groups were similar across the period 2004–2020, increases in disability have been slightly more pronounced in the social rented sector, especially since 2015. If these observed trends continue to 2030, then the overall adult age-standardized disability rate for those in the social rented sector would be 56%, almost double the figure of 29% in the owner-occupied sector. These future trends depend on unpredictable factors and the inherent uncertainty in these forecasts should be recognized, but nevertheless the results are a matter of concern for public health, public policy, and housing providers.

## Discussion

We have shown large, and increasing, differentials in the proportions reporting disability by housing tenure in the UK even after controlling for other indicators of socio-demographic status. Such differences in health and disability by housing tenure are important for planners and providers of housing and community services (43). For example if, as shown by these analyses, increasing proportions of social renters have long-term health problems, the costs of making housing adaptions may increase and the composition of housing types in local areas should be included in decisions about the siting of health facilities in recognition of the fact that demand for health services, especially mental health services, may vary substantially by housing tenure. The most substantial implications are for local employment markets, especially for older workers.

An important question unresolved in our analyses is what factors, other than the conventional sociodemographic characteristics we considered, might account for such substantial and increasing differentials. A likely major influence may be the increased weight over time attached to health status in allocation policies by social housing providers (68). There are also some factors relevant to health which we have been unable to control for. These include wealth which, although strongly related to educational level and occupational social class, may not be wholly captured by these indicators. Previous studies have found that wealth is associated with health, particularly in older age groups, with proposed mechanisms for the link including psychosocial benefits of a feeling of security, as well as enhanced ability to afford better living standards (86, 87). In addition to equity in their homes, older homeowners have greater average savings than private tenants, who in turn have more savings than social tenants, moreover in older age groups housing costs for owner occupiers may be lower if they have paid off their mortgage in comparison to tenants paying rent (68). We were also unable to take account of early life experiences which may be related to health, which is relevant since intergenerational continuities in housing tenure are substantial and are linked both to wealth and to housing conditions in childhood (70). We were also unable to consider neighborhood characteristics which studies have suggested are associated with health, particularly mental health (88) and are likely to be important, given the concentration of social housing in deprived areas (64). Related to neighborhood effects, a growing number of studies indicate that an individual’s mental health and health behaviors are influenced by those of people in their social network implying that concentrations of people with particular health related problems in neighborhoods may have ‘spillover’ effects (89).

Health differences between owners and social renting tenants are substantially larger in magnitude to those between, for example, partnered and unpartnered people or those with tertiary and those with no (or other) educational qualifications after controls for variables such as age. A number of reasons for the advantages of those living in owner-occupied as compared with those in rented properties have been identified. These include ontological security (36). The question arises as to whether such hypothesized explanations are sufficient to produce the differentials observed between owners and renters, especially with social renters. A complicating factor is the extent to which relationships are causal and, if so, how do they operate. Those in poor health, including those with disabling conditions, may require financial or instrumental assistance and move into rented accommodation and, in particular, social housing since they are deemed to be in need. In addition, those in poor health may be less able than others to move from the rented to the owner-occupied sector. If such direct causal effects of disability on housing tenure exist, the population-level effect would appear to be small because rates of movement between tenure groups are low: in England in 2019–2020, for example, eight thousand existing households were estimated to have moved from the owner occupied to the social rented sector, 0.05% of the 15.4 million households in the sector; movements in the opposite direction were too small to be reported [(90), Figure 1.10: Household moves, by tenure, 2019–2020]. Such moves could not account for the increasing divergence in reported disability up to the retirement ages or the convergence of disability rates at older ages. An alternative hypothesis is that housing experience affects the likelihood of being disabled, rather than the reverse. This would of course be controversial since housing access is strongly influenced by public policy, but if this had a strong influence, such effects might be expected to increase with duration in a particular housing tenure, but differentials tend to decrease rather than increase with duration (since tenure changes are infrequent at older ages).

In this paper, we cannot address the question of causality. One area we have discussed, partnership, has been recognized as strongly linked with health outcomes for centuries (91), but the relative contributions of selection into and out of partnership, and the economic, social and emotional benefits of partnership still remain unclear. These are the same sorts of questions that arise in examining the housing/health relationship. Selection into alternative tenures is substantial, with more advantaged groups being much more likely to be in the owner-occupied sector, but controlling for variables such as education, occupation and partnership accounts for only a limited part of overall observed differences. These selection effects have little influence at older rages since inter-tenure mobility is so low at these ages. The variables used here were largely fixed long before the age-group considered, apart from economic activity, which has a limited role as a determinant of disability status, since while there are reciprocal effects, there is a strong link from current disability status to current economic activity. The other variables considered, education, occupation and partnership, reflect past life course experiences often fixed in early adulthood, which are themselves determined by earlier experiences. The question of why two individuals with similar backgrounds based on these variables but differing only in their hosing tenure are likely to have substantially different levels of disability remains. Hypothesized explanations such as physical housing conditions, amenities and security are unlikely to wholly explain differences between tenures, especially between social renting and owner-occupied sectors since the most comprehensive set of indicators, such as the broad-based Decent Homes classification, indicate that standards are now better in the social rented than in other tenures.

Differentials in disability do not show clear cut patterns in that those cohorts which had high or low rates of home ownership do not appear to have consistent disability trends that match their housing experiences. Analyses using length of time at current address do not show any clear effect on disability status (length of residence does not, of course, give information about changes in housing tenure but only about place of residence so have not been discussed).

While the question of the relative contribution of the various factors to the substantial differentials observed between housing tenure groups remains, in part this is because any explanation would need to consider why the differentials rose sharply at later working ages as compared with younger and older age groups in the context of relatively low levels of mobility between sectors.

### Future prospects

While we have been mainly concerned with differences in disability between housing tenures, we recognize that there are major similarities as well. All cohorts to date have shown increasing rates of disability as they age, the fact that levels have been increasing at our baseline age of 40 means that the more recent cohorts in all tenure groups are likely to spend a higher proportion of their lives as disabled than todays’ older people.

Trends in housing tenure may be predicted with greater confidence than for many other variables because movement between sectors has historically been low (in the absence of large scale policy changes such as the sale of local authority housing to sitting tenants in the later part of the 20th century). The cohorts born around the early 1940s have higher rates of home ownership in recent decades than those born on either side of that period, in part due to the purchase of council homes noted above. However more recent cohorts now entering the Third Age with lower levels of home ownership are unlikely to change their situation in years to come.

The reduction in social housing provision has been particularly marked but inertia in the housing market means that the main effects are most apparent among younger adults. A consequence is that the private rented sector is becoming the only realistic option for a growing proportion of younger adults since access to the diminished supply of social housing is now prioritized to those with particular needs such as homelessness etc. and property price inflation has made ownership unaffordable for many. However, while this selection effect might be expected to have a strong effect on the health status of younger adults, this does not appear to be sufficient to account for the divergence in reported disability between tenure groups since around 2010 (Figure 3). Private rented accommodation has been unattractive as a long term housing option for many decades. However, it is highly likely that those aged 40 and over in 10 years’ time will be much less likely to be owners or social renters than is the case for those in recent decades. These are likely to be in the private rented sector, and face the prospect of lower average housing quality, much lower security (with current legislation) and high levels of rent increases.

Our results suggest that any strategy to addressing the challenges of an aging population and apparent increases in rates of disability in all age groups needs to pay attention to the role of housing and housing tenure. The disadvantages associated with poor housing have been acknowledged but policy recommendations have often been lacking. Three areas have received particular attention because of their direct or indirect links to physical and mental health; housing conditions, affordability, and security of tenure [(92), p. 93]. However, even though substantial efforts have been made to improve internal conditions in the social rented sector, health disparities between tenure groups have increased. The long lead times in the housing system means that the living conditions of the populations studied here were established in their early adulthood years, and increasingly young adults require substantial financial help from their home owning parents to themselves become owners further polarizing the distribution of tenure by wealth. Any substantial progress is likely to come from policies aimed at reducing inequalities in general, as well as policies to ensure housing supply and housing quality improve. In terms of research, a priority would be to address the role of neighborhood characteristics which we were unable to consider.

#### Justification

Most studies on the relationships between housing and health have been concerned with physical and environmental issues, such as the role of cold and damp. However, the level of disability (as legally defined in UK discrimination legislation) shows very substantial differences across a socially constructed variable, housing tenure. These differentials are shown not only to be large, but larger than more widely used measures of socio-economic position such as educational level. The relative and absolute disadvantages of those living in the social-rented sector have been increasing over time, and these remain after controlling for the socio-economic characteristics of those in the various sectors. These findings are linked to long-term politically driven changes. We use a much larger data source over an extended time period to provide robust evidence to support these findings that has not been possible hitherto. Analysis of these trends allows us to present projections to indicate how these trends may develop in years to come and to suggest that current trends are likely to exacerbate current problems.

## Data availability statement

The datasets presented in this article are not readily available because the data analyzed in this study is Crown Copyright and subject to license from the UK Office for National Statistics that does not allow the distribution of the data. Requests to access these datasets should be directed to the UK Data Archive https://www.data-archive.ac.uk/.

## Ethics statement

The studies involving humans were approved by University of Essex Ethics Committee. The studies were conducted in accordance with the local legislation and institutional requirements. The participants provided their written informed consent to participate in this study.

## Author contributions

EG and MM conceived and drafted the manuscript. MM undertook the statistical analysis. All authors contributed to the article and approved the submitted version.

## Footnotes

^1^A 10 per cent home ownership figure for 1914 is widely cited [e.g., (59), p. 12], but revised published analysis suggest a higher figure.

^2^In 1986, a new Housing Act raised discounts for flats to between 44 per cent (after 2 years of tenancy) and 70 per cent (after 15 years). Discounts for houses were 32 per cent after 2 years’ tenancy, increasing to 60 per cent after 30 years (66).
