# Supplementary figures and images for: Housing tenure and disability in the UK: trends and projections 2004–2030
Source: Front Public Health. 2024 Jan 4;11:1248909. doi: 10.3389/fpubh.2023.1248909 (PMC10795505; doi:10.3389/fpubh.2023.1248909)

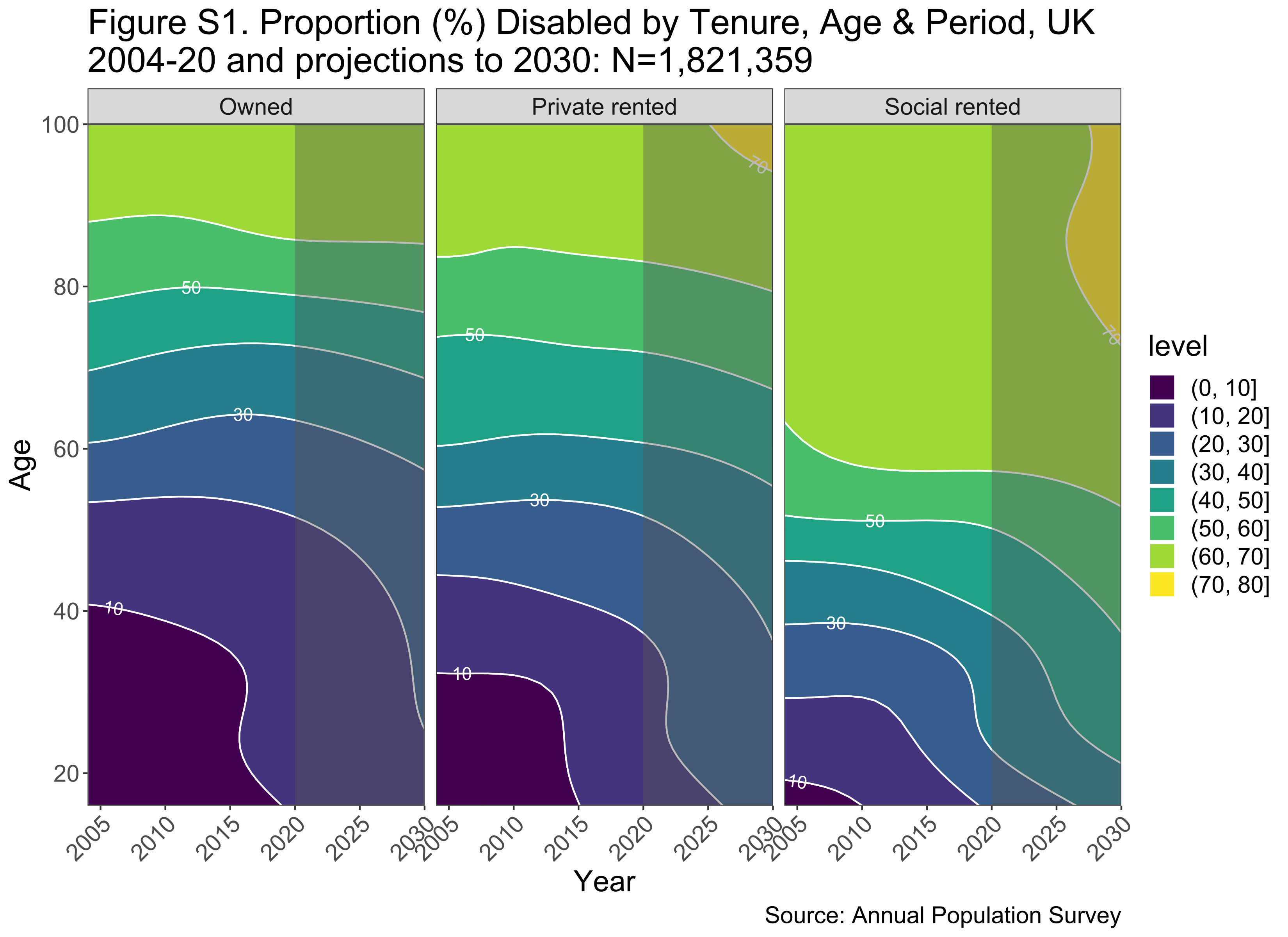

Supplement: Supplementary file 1 [file Image_1.png]
