# Supplementary material for: Housing tenure and disability in the UK: trends and projections 2004–2030
Source: Front Public Health. 2024 Jan 4;11:1248909. doi: 10.3389/fpubh.2023.1248909 (PMC10795505; doi:10.3389/fpubh.2023.1248909)

**Table S1. Odds ratios for models: single variable models include: single covariate, sex and age; Joint variable model includes all covariates, age and sex (see Figure 1)**

| Covariate | Variable | Single variable |  |  | Joint variable |  |  |
| --- | --- | --- | --- | --- | --- | --- | --- |
|  |  | OR | *2.50%* | *97.50%* | OR | *2.50%* | *97.50%* |
| Tenure | Private rented | 1.80 | *1.75* | *1.84* | 1.30 | *1.25* | *1.34* |
| **Tenure** | **Social rented** | **4.47** | ***4.38*** | ***4.56*** | **1.95** | ***1.90*** | ***2.01*** |
| **Marital** | **Divorced/Separated** | **1.83** | ***1.79*** | ***1.87*** | **1.32** | ***1.28*** | ***1.35*** |
| Marital | Single, never married | 1.91 | *1.87* | *1.95* | 1.18 | *1.15* | *1.21* |
| Marital | Widowed | 1.70 | *1.63* | *1.77* | 1.18 | *1.12* | *1.23* |
| HiQual | Upper secondary | 1.45 | *1.42* | *1.49* | 1.15 | *1.12* | *1.19* |
| HiQual | Lower secondary | 1.58 | *1.54* | *1.61* | 1.12 | *1.09* | *1.15* |
| **HiQual** | **Other/None** | **2.62** | ***2.56*** | ***2.67*** | **1.24** | ***1.20*** | ***1.27*** |
| NS-SEC | Medium | 1.41 | *1.37* | *1.44* | 1.19 | *1.16* | *1.22* |
| **NS-SEC** | **Low** | **1.91** | ***1.86*** | ***1.95*** | **1.26** | ***1.22*** | ***1.30*** |
| NS-SEC | Never worked, unemployed, and nec | 6.13 | *5.98* | *6.29* | 1.82 | *1.76* | *1.88* |
| EconAct | Other not working | 1.93 | *1.89* | *1.97* | 1.52 | *1.48* | *1.56* |
| **EconAct** | **Sick/injured/disabled** | **111.47** | ***104.62*** | ***118.90*** | **61.67** | ***57.46*** | ***66.27*** |

Note: Largest value within each covariate in bold. Approximate 95% C.I. also shown.

Source: Annual Population Survey


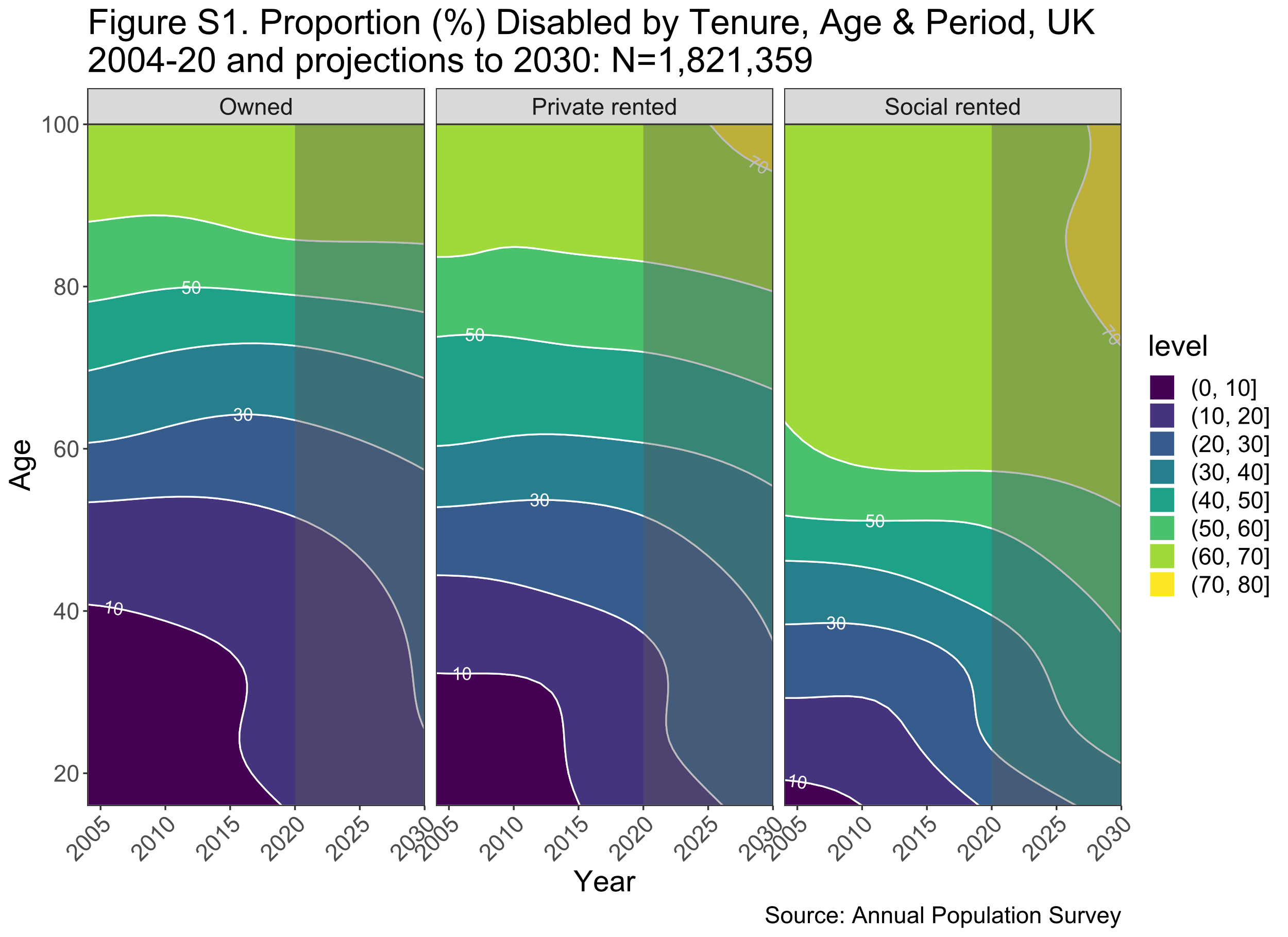

Supplement: Supplementary file 2 [file Table_1.DOCX]
