# Supplementary material for: Housing tenure and disability in the UK: trends and projections 2004–2030
Source: Front Public Health. 2024 Jan 4;11:1248909. doi: 10.3389/fpubh.2023.1248909 (PMC10795505; doi:10.3389/fpubh.2023.1248909)
Supplement: Supplementary file 3 [file Table_2.DOCX]

**Table S1. Odds ratios for model of Figure 1, logistic regression model for disability sex (see Figure 1)**

|  | **Disability** | | | |
| --- | --- | --- | --- | --- |
| *Predictors* | *Odds Ratios* | *Standard Error* | *95% CI* | *p* |
| **Tenure**: Owned | *Reference* |  |  |  |
| Private rented | 1.29 | 0.02 | 1.25 – 1.34 | <0.001 |
| Social rented | 1.96 | 0.03 | 1.90 – 2.02 | <0.001 |
|  |  |  |  |  |
| **Sex**: Male | *Reference* |  |  |  |
| Female | 1.18 | 0.01 | 1.16 – 1.21 | <0.001 |
|  |  |  |  |  |
| **HiQual**: Tertiary | *Reference* |  |  |  |
| Upper secondary | 1.16 | 0.02 | 1.12 – 1.19 | <0.001 |
| Lower secondary | 1.12 | 0.02 | 1.09 – 1.16 | <0.001 |
| Other/None | 1.24 | 0.02 | 1.20 – 1.27 | <0.001 |
|  |  |  |  |  |
| **NSEC**: Managerial and professional | *Reference* |  |  |  |
| Intermediate occupations | 1.12 | 0.02 | 1.08 – 1.16 | <0.001 |
| Small employers and own account workers | 1.30 | 0.02 | 1.25 – 1.35 | <0.001 |
| Lower supervisory and technical | 1.13 | 0.03 | 1.08 – 1.18 | <0.001 |
| Routine and semi-routine | 1.26 | 0.02 | 1.22 – 1.30 | <0.001 |
| Never worked, unemployed, and nec | 1.82 | 0.03 | 1.76 – 1.88 | <0.001 |
|  |  |  |  |  |
| **EconAct**: In employment | *Reference* |  |  |  |
| ILO unemployed | 1.71 | 0.05 | 1.61 – 1.82 | <0.001 |
| Sick/Injured/Disabled | 61.91 | 2.25 | 57.69 – 66.53 | <0.001 |
| Retired | 1.57 | 0.03 | 1.52 – 1.62 | <0.001 |
| Other (unemp, homemaker etc.) | 1.42 | 0.03 | 1.37 – 1.47 | <0.001 |
|  |  |  |  |  |
| **Marita**l: Married | *Reference* |  |  |  |
| Divorced/Separated | 1.31 | 0.02 | 1.28 – 1.35 | <0.001 |
| Single, never married | 1.17 | 0.02 | 1.14 – 1.21 | <0.001 |
| Widowed | 1.17 | 0.03 | 1.12 – 1.23 | <0.001 |

Observations 281,219; R2 Nagelkerke 0.252;AIC 261,727.50;log-Likelihood -13,081,843.75

Source: Annual Population Survey

**Table S2. Odds ratios for logistic regression models for disability: single variable models include: single covariate, sex and age; Joint variable model includes all covariates, age and sex (see Figure 1)**

| *Covariate* | *Variable* | *Single variable model* | | | *Joint variable model* | | |
| --- | --- | --- | --- | --- | --- | --- | --- |
|  |  | *OR* | *2.50% CII* | *97.50% CI* | *OR* | *2.50% CI* | *97.50% CI* |
| Tenure | Private rented | 1.80 | *1.75* | *1.84* | 1.30 | *1.25* | *1.34* |
| **Tenure** | **Social rented** | **4.47** | ***4.38*** | ***4.56*** | **1.95** | ***1.90*** | ***2.01*** |
|  |  |  |  |  |  |  |  |
| **Marital** | **Divorced/Separated** | **1.83** | ***1.79*** | ***1.87*** | **1.32** | ***1.28*** | ***1.35*** |
| Marital | Single, never married | 1.91 | *1.87* | *1.95* | 1.18 | *1.15* | *1.21* |
| Marital | Widowed | 1.70 | *1.63* | *1.77* | 1.18 | *1.12* | *1.23* |
|  |  |  |  |  |  |  |  |
| HiQual | Upper secondary | 1.45 | *1.42* | *1.49* | 1.15 | *1.12* | *1.19* |
| HiQual | Lower secondary | 1.58 | *1.54* | *1.61* | 1.12 | *1.09* | *1.15* |
| **HiQual** | **Other/None** | **2.62** | ***2.56*** | ***2.67*** | **1.24** | ***1.20*** | ***1.27*** |
|  |  |  |  |  |  |  |  |
| NS-SEC | Medium | 1.41 | *1.37* | *1.44* | 1.19 | *1.16* | *1.22* |
| NS-SEC | Low | 1.91 | *1.86* | *1.95* | 1.26 | *1.22* | *1.30* |
| **NS-SEC** | **Never worked, unemployed, and nec** | **6.13** | ***5.98*** | ***6.29*** | **1.82** | ***1.76*** | ***1.88*** |
|  |  |  |  |  |  |  |  |
| EconAct | Other not working | 1.93 | *1.89* | *1.97* | 1.52 | *1.48* | *1.56* |
| **EconAct** | **Sick/injured/disabled** | **111.47** | ***104.62*** | ***118.90*** | **61.67** | ***57.46*** | ***66.27*** |

Note: Largest value within each covariate in bold. Approximate 95% C.I. also shown.

Source: Annual Population Survey

**Table S3. EMM Predicted values (percent) based on logistic regression for disability (see Figure 2)**

| *Housing type* | *Socio-demographic variable* | *EMM predicted* | *95% CI low* | *95% CI high* |
| --- | --- | --- | --- | --- |
| *Tenure* | *NSEC* |  |  |  |
| Owned | High | 18 | 17 | 18 |
| Owned | Medium | 21 | 20 | 21 |
| Owned | Low | 24 | 23 | 24 |
| Private rented | High | 22 | 21 | 23 |
| Private rented | Medium | 27 | 26 | 28 |
| Private rented | Low | 29 | 28 | 30 |
| Social rented | High | 37 | 36 | 39 |
| Social rented | Medium | 38 | 37 | 39 |
| Social rented | Low | 39 | 39 | 40 |
|  |  |  |  |  |
| *Tenure* | *HiQual* |  |  |  |
| Owned | Tertiary | 24 | 24 | 25 |
| Owned | Secondary | 28 | 28 | 29 |
| Owned | Other/None | 33 | 32 | 33 |
| Private rented | Tertiary | 29 | 28 | 30 |
| Private rented | Secondary | 38 | 37 | 39 |
| Private rented | Other/None | 40 | 38 | 41 |
| Social rented | Tertiary | 48 | 47 | 50 |
| Social rented | Secondary | 52 | 51 | 52 |
| Social rented | Other/None | 54 | 53 | 55 |
|  |  |  |  |  |
| *Tenure* | *Partner* |  |  |  |
| Owned | Partnered | 24 | 24 | 25 |
| Owned | Not partnered | 31 | 31 | 32 |
| Private rented | Partnered | 30 | 29 | 31 |
| Private rented | Not partnered | 42 | 41 | 43 |
| Social rented | Partnered | 44 | 43 | 45 |
| Social rented | Not partnered | 57 | 56 | 58 |
|  |  |  |  |  |
| *Tenure* | *EconAct* |  |  |  |
| Owned | In employment | 20 | 20 | 20 |
| Owned | Other not working | 26 | 25 | 26 |
| Owned | Sick/Injured/Disabled | 94 | 94 | 95 |
| Private rented | In employment | 23 | 22 | 24 |
| Private rented | Other not working | 35 | 34 | 37 |
| Private rented | Sick/Injured/Disabled | 95 | 94 | 96 |
| Social rented | In employment | 29 | 28 | 30 |
| Social rented | Other not working | 46 | 45 | 47 |
| Social rented | Sick/Injured/Disabled | 96 | 96 | 97 |

Source: Annual Population Survey
