# Supplementary material for: Housing tenure and disability in the UK: trends and projections 2004–2030
Source: Front Public Health. 2024 Jan 4;11:1248909. doi: 10.3389/fpubh.2023.1248909 (PMC10795505; doi:10.3389/fpubh.2023.1248909)
Supplement: Supplementary file 4 [file Table_2.pdf]

Table S21. Odds ratios for model of Figure 1, logistic regression model for disability sex (see Figure 1)

	Disability			
Predictors	*Odds Ratios*	*Standard Error*	*95% CI*	*p*
**Tenure**: Owned	*Reference*			
Private rented	1.29	0.02	1.25 – 1.34	<0.001
Social rented	1.96	0.03	1.90 – 2.02	<0.001
**Sex**: Male	*Reference*			
Female	1.18	0.01	1.16 – 1.21	<0.001
**HiQual**: Tertiary	*Reference*			
Upper secondary	1.16	0.02	1.12 – 1.19	<0.001
Lower secondary	1.12	0.02	1.09 – 1.16	<0.001
Other/None	1.24	0.02	1.20 – 1.27	<0.001
**NSEC**: Managerial and professional	*Reference*			
Intermediate occupations	1.12	0.02	1.08 – 1.16	<0.001
Small employers and own account workers	1.30	0.02	1.25 – 1.35	<0.001
Lower supervisory and technical	1.13	0.03	1.08 – 1.18	<0.001
Routine and semi-routine	1.26	0.02	1.22 – 1.30	<0.001
Never worked, unemployed, and nec	1.82	0.03	1.76 – 1.88	<0.001
**EconAct**: In employment	*Reference*			
ILO unemployed	1.71	0.05	1.61 – 1.82	<0.001
Sick/Injured/Disabled	61.91	2.25	57.69 – 66.53	<0.001
Retired	1.57	0.03	1.52 – 1.62	<0.001
Other (unemp, homemaker etc.)	1.42	0.03	1.37 – 1.47	<0.001
**Marital**: Married	*Reference*			
Divorced/Separated	1.31	0.02	1.28 – 1.35	<0.001
Single, never married	1.17	0.02	1.14 – 1.21	<0.001
Widowed	1.17	0.03	1.12 – 1.23	<0.001

Observations 281,219; R2 Nagelkerke 0.252;AIC 261,727.50;log-Likelihood -13,081,843.75

Source: Annual Population Survey

Table S2. Odds ratios for logistic regression models for disability: single variable models include: single covariate, sex and age; Joint variable model includes all covariates, age and sex (see Figure 1)

Covariate	*Variable*	*Single variable model*			*Joint variable model*		
		OR	*2.50% CII*	*97.50% CI*	*OR*	*2.50% CI*	*97.50% CI*
Tenure	Private rented	1.80	*1.75*	*1.84*	1.30	*1.25*	*1.34*
**Tenure**	**Social rented**	**4.47**	***4.38***	***4.56***	**1.95**	***1.90***	***2.01***
**Marital**	**Divorced/Separated**	**1.83**	***1.79***	***1.87***	**1.32**	***1.28***	***1.35***
Marital	Single, never married	1.91	*1.87*	*1.95*	1.18	*1.15*	*1.21*
Marital	Widowed	1.70	*1.63*	*1.77*	1.18	*1.12*	*1.23*
HiQual	Upper secondary	1.45	*1.42*	*1.49*	1.15	*1.12*	*1.19*
HiQual	Lower secondary	1.58	*1.54*	*1.61*	1.12	*1.09*	*1.15*
**HiQual**	**Other/None**	**2.62**	***2.56***	***2.67***	**1.24**	***1.20***	***1.27***
NS-SEC	Medium	1.41	*1.37*	*1.44*	1.19	*1.16*	*1.22*
NS-SEC	Low	1.91	*1.86*	*1.95*	1.26	*1.22*	*1.30*
**NS-SEC**	**Never worked, unemployed, and nec**	**6.13**	***5.98***	***6.29***	**1.82**	***1.76***	***1.88***
EconAct	Other not working	1.93	*1.89*	*1.97*	1.52	*1.48*	*1.56*
**EconAct**	**Sick/injured/disabled**	**111.47**	***104.62***	***118.90***	**61.67**	***57.46***	***66.27***

Note: Largest value within each covariate in bold. Approximate 95% C.I. also shown.

Source: Annual Population Survey

Table S3. EMM Predicted values (percent) based on logistic regression for disability (see Figure 2)

Housing type	*Socio-demographic variable*	*EMM predicted*	*95% CI low*	*95% CI high*
Tenure	*NSEC*			
Owned	High	18	17	18
Owned	Medium	21	20	21
Owned	Low	24	23	24
Private rented	High	22	21	23
Private rented	Medium	27	26	28
Private rented	Low	29	28	30
Social rented	High	37	36	39
Social rented	Medium	38	37	39
Social rented	Low	39	39	40
Tenure	*HiQual*			
Owned	Tertiary	24	24	25
Owned	Secondary	28	28	29
Owned	Other/None	33	32	33
Private rented	Tertiary	29	28	30
Private rented	Secondary	38	37	39
Private rented	Other/None	40	38	41
Social rented	Tertiary	48	47	50
Social rented	Secondary	52	51	52
Social rented	Other/None	54	53	55
Tenure	*Partner*			
Owned	Partnered	24	24	25
Owned	Not partnered	31	31	32
Private rented	Partnered	30	29	31
Private rented	Not partnered	42	41	43
Social rented	Partnered	44	43	45
Social rented	Not partnered	57	56	58
Tenure	*EconAct*			
Owned	In employment	20	20	20
Owned	Other not working	26	25	26
Owned	Sick/Injured/Disabled	94	94	95
Private rented	In employment	23	22	24
Private rented	Other not working	35	34	37
Private rented	Sick/Injured/Disabled	95	94	96
Social rented	In employment	29	28	30
Social rented	Other not working	46	45	47
Social rented	Sick/Injured/Disabled	96	96	97

Source: Annual Population Survey
